# Meta-analysis of the role of *IL-6* rs1800795 polymorphism in the susceptibility to prostate cancer: Evidence based on 17 studies

**DOI:** 10.1097/MD.0000000000006126

**Published:** 2017-03-24

**Authors:** Tong-Zu Liu, Zhong-Qiang Guo, Ting Wang, Yue Cao, Di Huang, Xing-Huan Wang

**Affiliations:** a Department of Urology; b Center for Evidence-Based and Translational Medicine, Zhongnan Hospital of Wuhan University, Wuhan, China.

**Keywords:** cytokine, immune response, interleukin-6, prostate cancer, susceptibility

## Abstract

Playing critical roles in immune responses, interleukin-6 (IL-6) has been proposed to be involved in the development of multiple cancers, including prostate cancer. The rs1800795 polymorphism in the promoter of the gene IL-6 can affect the transcription and expression of the gene, becoming a common target in association studies on tumors. We therefore carried out this meta-analysis to further discuss the relationship of this polymorphism with the risk of prostate cancer.

Relevant publications were retrieved from the electronic databases. The strength of the correlation between *IL-6* rs1800795 polymorphism and prostate cancer risk was evaluated using pooled odds ratios (ORs) with their 95% confidence intervals (95% CIs). *Q* test was adopted to examine between-study heterogeneity, with *P* < 0.05 as significant level. Subgroup and meta-regression analyses were conducted to explore potential source of heterogeneity. Sensitivity analysis was implemented to test the statistical stability of the final results. In addition, funnel plot and Egger test were employed to inspect publication bias among included studies.

A total of 13 132 cases and 15 282 controls were ultimately incorporated into the present study. Overall estimates revealed no significant relationship between *IL-6* rs1800795 polymorphism and prostate cancer risk in total analysis, but a risk-increasing effect of the polymorphism was detected in African-American subgroup under CC versus GG and CC versus GG + GC contrasts (OR 3.43, 95% CI 1.01–11.71; OR 3.51, 95% CI 1.04–11.82) after subgroup analysis by ethnicity.

*IL-6* rs1800795 polymorphism may enhance the susceptibility to prostate cancer in African-American men.

## 1 Introduction

Prostate cancer is 1 of the most commonly diagnosed male malignancies jeopardizing their health, and frequently affects those aged over 60 years.^[1]^ In 2012, this cancer caused 1.1 million new cases and 0.3 million deaths around the world, ranking the second and fifth positions in terms of morbidity and mortality, respectively.^[2]^ The incidence rate of this cancer varies among regions and races, with about two-third of total prostate cancer cases occurring among men in more developed countries who only account for just 17% of global male population.^[3]^ This malignancy, however, still shows an upward tendency in its incidence in both previously high-risk regions and in relatively low-risk ones during the past few decades.^[4]^ At the moment, the exact etiology of prostate cancer is still poorly understood, but some aspects have been confirmed as risk factors for it, such as age, race, and family history.^[5]^ In addition, the differences of incidence rate between races and regions may be related to lifestyles, environmental conditions, and genetic backgrounds.^[6]^ Existing documents have demonstrated that among material bases for genetic susceptibility to prostate cancer, single-nucleotide polymorphism (SNP) presents a key one.^[7]^

Interleukin-6 (IL-6), a bioactive peptide with multiple functions, is mainly originated from mononuclear phagocytes and partly from fibroblasts, T and B lymphocytes, and vascular endothelial cells.^[8,9]^ Human *IL-6* gene is located at chromosome 7p21–14 with a total length of 5 kb, and contains 4 introns and 5 exons.^[10]^ With the advancement of studies on tumor pathogenesis, the initiation and progression of tumors have been uncovered to be closely related to the loss of regulation of host immune system on tumors.^[11]^ Cytokines play key regulating roles in immune responses,^[12]^ and some of them even directly influence the growth and invasion of tumors. Reportedly, IL-6, as an autocrine or paracrine factor, is able to not only regulate tumor growth via straight effects on tumor cells, but also indirectly promote the growth of tumor cells through affecting host environments, such as inducing antiapoptosis, neovascularization, and acute phase responses.^[13,14]^ In recent years, the role of IL-6 in the origination and development of malignant tumors has been kept exploring, and abnormal expression of this cytokine has been observed in multiple tumor tissues, such as myeloma,^[15]^ renal cancer,^[16]^ hepatocellular carcinoma,^[17]^ lung cancer,^[18]^ and esophageal carcinoma.^[19]^

The rs1800795 polymorphism in the promoter of the coding gene *IL-6* can affect the transcription of the gene, and thus alter the cytokine production.^[20]^ Therefore, this SNP has been discussed in previous association studies on various cancers. In this meta-analysis, we targeted this polymorphism to examine its role in the susceptibility to prostate cancer.

## 2 Methods

### 2.1 Literature retrieval

This meta-analysis was conducted in accordance with the checklist of the Meta-analysis of Observational Studies in Epidemiology (MOOSE) guidelines. A systemic literature search was conducted in the databases PubMed, EMBASE, Goggle Scholar, and CNKI using the combination of the following terms: “prostate” or “prostatic,” “cancer” or “carcinoma” or “tumor” or “neoplasm” or “malignancy” or “malignant,” “interleukin-6” or “IL-6” or “ BSF2” or “HGF” or “HSF,” and “polymorphism” or “mutation” or “SNP” or “variant” or “polymorphisms.” To supplement the yield of database searching, we also manually checked the reference lists of relevant papers.

### 2.2 Inclusion and exclusion criteria

Studies focused on the relationship between *IL-6* rs1800795 polymorphism and prostate cancer risk might be qualified for our meta-analysis, and could enter into next circle of eligibility assessment; otherwise, they were excluded from the present study. Predesigned criteria for detailed evaluation contained the following aspects: with a case-control design; stating sufficient information on genotype and/or allele frequencies in cases and controls; and concerning human beings. Articles failing to satisfy any one of the above standards were removed in this course. As for reports with overlapping data, the one would be finally selected which covered the most comprehensive information.

### 2.3 Data extraction

Two reviewers took charge of data extraction from all eligible studies, and completed cross-check on the recorded data to guarantee their accuracy. If disagreements occurred in this process, they would be settled through discussion between these 2 reviewers until consensus was achieved on each item. Many aspects of included studies were extracted, including first author's name, year of publication, country of origin, ethnic line, control source, genotyping method, genotype and/or allele frequencies in case and control groups, and *P* value for Hardy–Weinberg equilibrium (HWE) in controls.

### 2.4 Statistical analysis

STATA 12.0 software (Stata Corporation, College Station, TX) was utilized to complete all data syntheses in this meta-analysis. The intensity of the relationship between *IL-6* rs1800795 polymorphism and the susceptibility to prostate cancer was appraised through calculating pooled odds ratios (ORs) with the corresponding 95% confidence intervals (95% CIs). Heterogeneity between selected studies was inspected using chi-square-based *Q* test, with *P* value more or less than 0.05, representing the absence or presence of significant heterogeneity. When significant heterogeneity existed, we would implement meta-regression analysis to identify its possible source. When *P* > 0.05 in *Q* test, fixed-effects model was chosen for calculating summary ORs; or else, random-effects model was applied. Moreover, subgroup analyses, which were stratified according to the patients’ ethnicity (Asia, Caucasian, African-American, and mixed groups), and source of controls (hospital, population, and NA groups) were performed to explore potential sources of heterogeneity and the differences among them. In addition, sensitivity analysis was conducted through sequential deleting each of included studies so as to verify the stability of overall estimates. Publication bias across enrolled studies was investigated with both Begg funnel plot and Egger regression test.

## 3 Results

### 3.1 Outcome of literature selection and study characteristics

**Figure 1:**
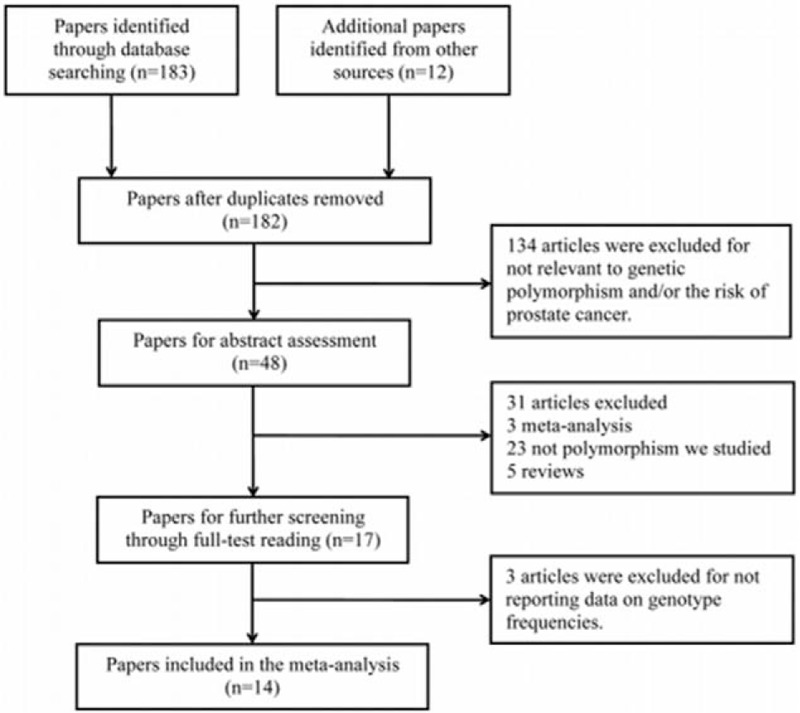
Flowchart of literature selection with detailed reasons for exclusion.

In the initial search, 195 potentially relevant publications were identified, and then 13 duplicates were excluded from the current meta-analysis (Fig. 1). In further eligibility assessment, 168 more articles were removed for not relevant to genetic polymorphism or prostate cancer risk (n = 134), meta-analyses (n = 3), not focusing on our studied SNP (n = 23), reviews (n = 5), and insufficient data (n = 3). As a consequence, a total of 14 eligible reports containing 17 studies were ultimately incorporated into this meta-analysis,^[21–34]^ enrolling 13,132 cases and 15,282 controls. Table 1 lists the main characteristics of all included studies.

**Table 1:**
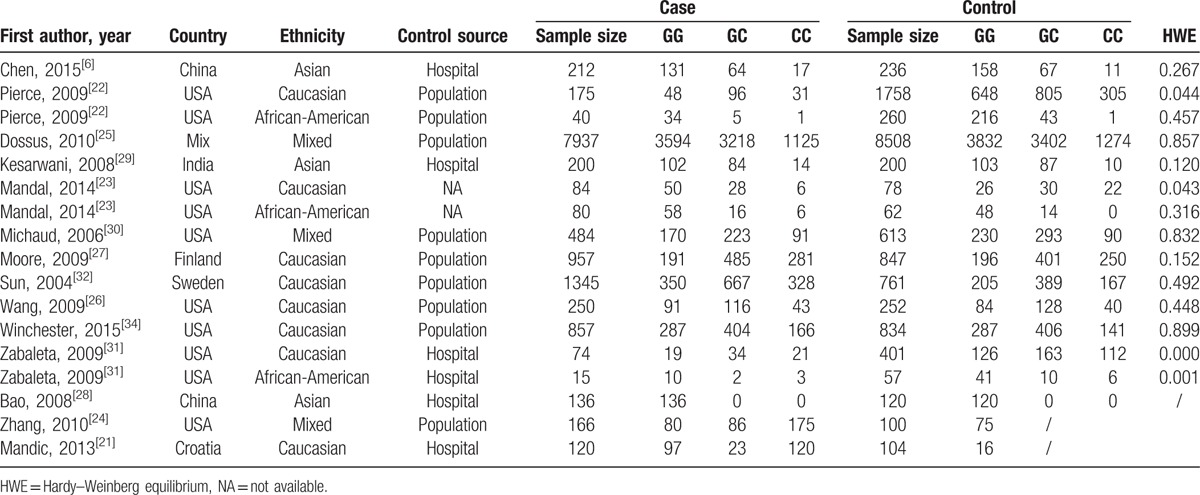
Essential information of included studies in the meta-analysis.

### 3.2 Quantitative data synthesis

**Figure 2:**
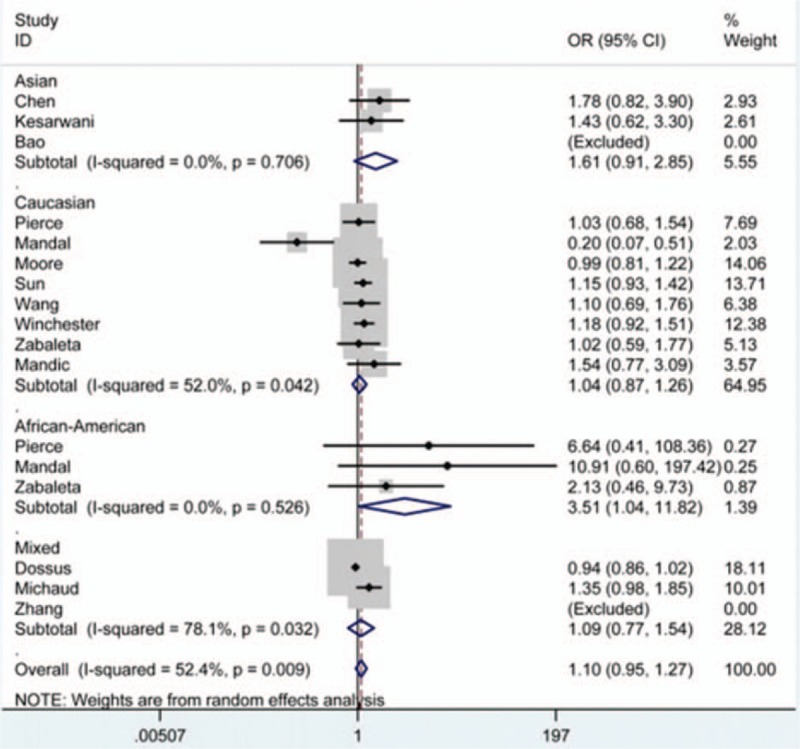
Prostate cancer risk associated with *IL-6* rs1800795 polymorphism under CC versus GG + GC contrast after stratification analysis by ethnicity.

As shown in Table 2, *IL-6* rs1800795 polymorphism showed an increasing effect on the risk of prostate cancer in total analysis, but such influence had no statistical significance, even in stratified analysis by control source. However, after stratification analysis by ethnicity, it significantly elevated the cancer risk in African-American subgroup under CC versus GG and CC versus GG + GC (Fig. 2) genetic models (OR 3.43, 95% CI 1.01–11.71; OR 3.51, 95% CI 1.04–11.82).

**Table 2:**
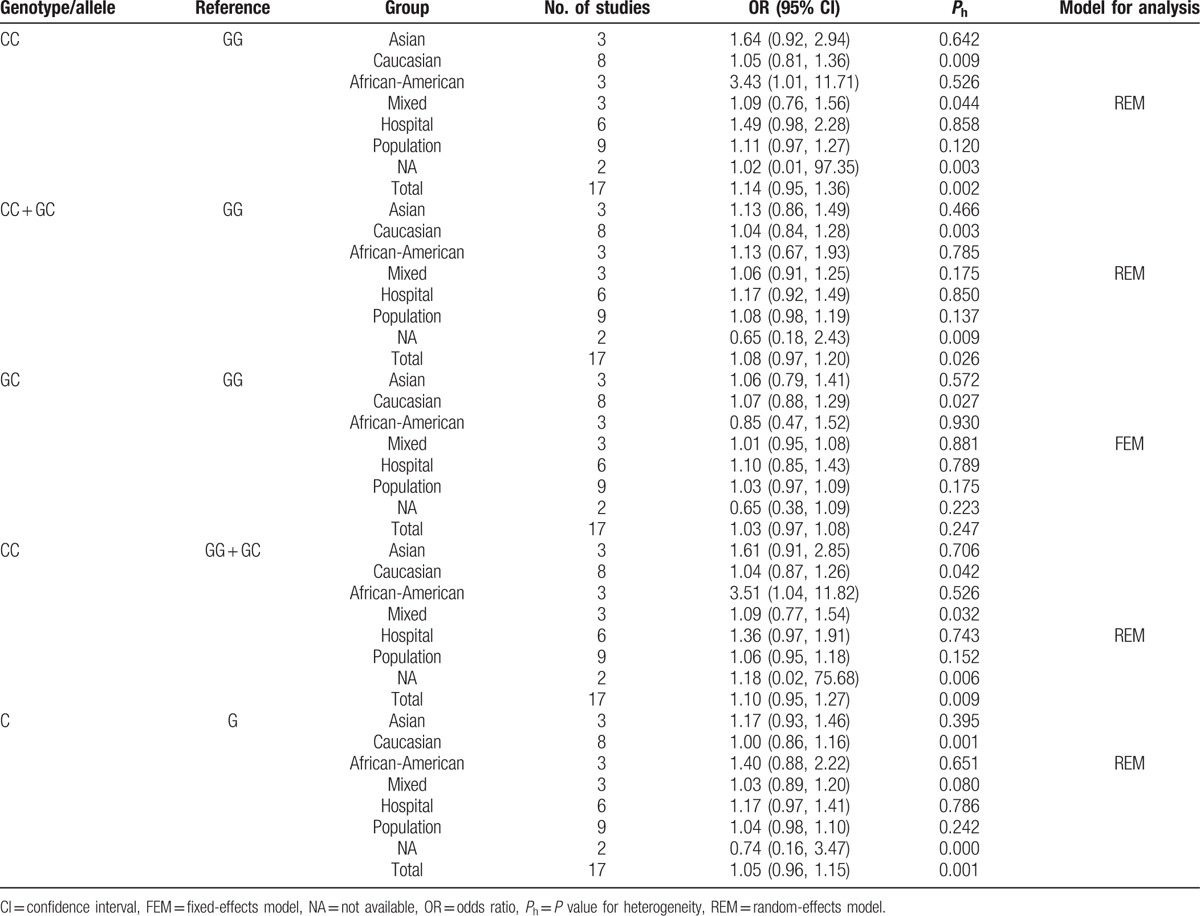
Association between *IL-6* rs1800795 polymorphism and prostate cancer susceptibility.

### 3.3 Heterogeneity test

Significant heterogeneity was detected among included studies under 4 contrasts: CC versus GG, CC + GC versus GG, CC versus GG + GC, and C versus G; so the random-effects model was engaged in calculating pooled ORs under these cases. To identify the source of the heterogeneity, we also implemented meta-regression analysis, and the results (data not shown) manifested that such heterogeneity could be mainly attributed to the study by Mandal et al.^[23]^

Under the other 1 comparison, the fixed-effects model was chosen for OR calculation in view of the absence of significant heterogeneity.

### 3.4 Sensitivity analysis

We recalculated summary ORs after expunging each of the eligible studies in turn, and then compared them with original overall estimates. The comparison detected no qualitative alteration between the effects, verifying that our results were statistical robust and stable.

### 3.5 Publication bias investigation

**Figure 3:**
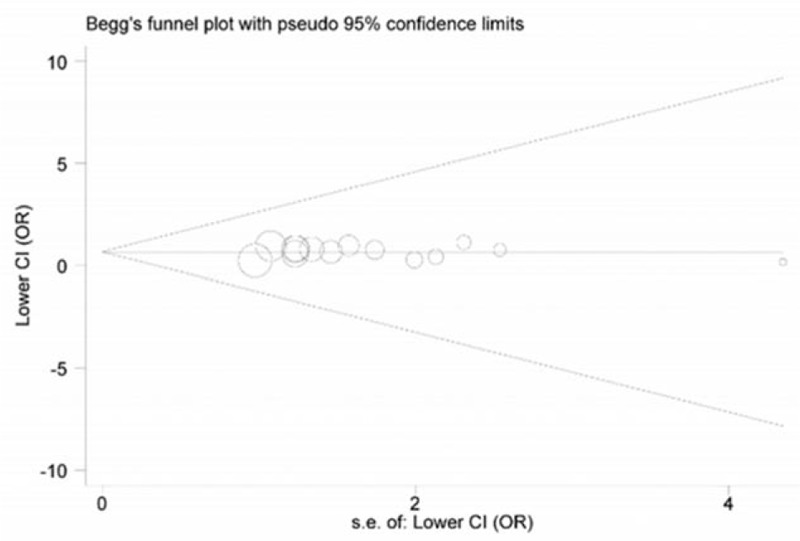
Begg funnel plot for publication bias.

In visual inspection of Begg funnel plots, we found the shape of these plots seemed symmetrical (Fig. 3). Moreover, the statistical data from Egger test further confirmed such symmetry (*P* = 0.879). Therefore, publication bias across the selected studies in this meta-analysis was negligible.

## 4 Discussion

Up to now, the etiology of prostate cancer is still unclear, and relevant researches have put forward multiple potentially relevant aspects, involving environmental factors and genetic factors. The polymorphism, as inherent mechanisms of human diseases, has been used to explain the differences in incidence, clinical manifestations, and response to treatment of tumor patients, including prostate cancer.^[28]^ Previous studies have shown that the *IL-6* polymorphism (rs1800795) may predispose to prostate cancer and influence disease severity.^[23,29]^ The rs1800795 polymorphism located in the promoter of the coding gene *IL-6*, has been reported to affect its gene transcription and thus change serum IL-6 levels.

Evidences exhibit that IL-6 plays an important role in the transformation of prostate cancer from hormone-dependent to hormone-independent, thus attenuating the efficiency of endocrine therapy.^[35]^ As a pleiotropic cytokine, IL-6 can regulate many cell functions, including immune defensive mechanism, cell proliferation, and differentiation, and also the production of haemocytes. In addition, it also has close relationship with the origination and progression of multiple tumors, affecting the development of tumors through influencing cells’ ability of adhesion and activity, the formation of thrombi, the expression of tumor-specific antigens, and the proliferation of tumor cells.^[36–38]^ Several studies have shown an association between *IL-6* gene polymorphisms and the risk of prostate cancer, but the results are inconclusive. For example, Mandal et al,^[23]^ in their study, found different effects of this polymorphism on the risk of prostate cancer. Specifically, the GG genotype of the polymorphism increased the risk in Caucasian subjects, whereas the CC genotype displayed a similar trend in African-American cases. In other words, *IL-6* -174G>C polymorphism might play totally opposite effects in different races.

To statistically discuss this relationship, we designed the present meta-analysis based on previously published studies on this topic. After data syntheses, we found no significant correlation of *IL-6* rs1800795 polymorphism with the susceptibility to prostate cancer in total analysis. However, the polymorphism significantly elevated the risk in African-American group after stratification analysis by ethnicity under CC versus GG and CC versus GG + GC contrasts, which was consistent with the findings from the study by Mandal et al. In other subgroups, no significant relationship was detected. Generally, the findings from this meta-analysis were relatively reliable because they not only were obtained on the basis of a larger sample size of 13,132 cases and 15,282 controls, but also were tested through some examinations. However, significant heterogeneity was detected among included studies, and the results of meta-regression analysis manifested that such heterogeneity could be mainly attributed to the study by Mandal et al. So the random-effects model was engaged in calculating pooled ORs under these cases. Moreover, sensitivity analysis was conducted by expunging each of the eligible studies in turn to see whether a particular omission could influence the overall estimates. The overall estimates expressed no qualitative change after the deletion of the study by Mandal et al, and similar results were observed after removal of other included studies during the sensitivity analysis, confirming that our results were statistical robust and stable. As for possible reasons for the study by Mandal et al presenting the source of significant heterogeneity, we speculated that their finding about the opposite effects of *IL-6* rs1800795 polymorphism on prostate cancer risk in 2 different descent groups itself might partly contribute to the occurrence of the heterogeneity. In the present meta-analysis, limited number of patients in various ethnicity groups and different sources of controls may contribute to relatively extended CIs. Future studies that include a larger number of patients with better study designs need to be conducted to clarify this important issue. When it came to publication bias, neither funnel plots nor statistical data from Egger test supported the existence of significant bias. Based on these tests, we believed our conclusion had certain strength.

That having been said, the results from the current meta-analysis still should be applied warily due to some inevitable limitations in our study. First of all, we only selected eligible articles from those previously published, so some relevant papers unpublished might be missed, generating certain publication bias, though not detected even with funnel plot or Egger test. Apart from this, detailed subgroup analyses were not performed according to important factors involved in the risk of prostate cancer, such as age, smoking status, alcohol consumption, and family history, owing to restricted information in original articles. Hence, the final effects from this meta-analysis might have some bias to a certain degree. Furthermore, plausible effects of gene–gene and gene–environment interactions on the cancer risk were not taken into account because of limited information as well.

In summary, *IL-6* rs1800795 polymorphism may not have an independent influence on the susceptibility to prostate cancer in general population, but it may significantly increase the risk in African-American males. In view of the above mentioned restrictions, these results need to be further verified by studies fully considering the effects of major factors involved in the cancer incidence.
